# Factors influencing health workers’ adherence to malaria treatment guidelines in under-five children in Nigeria: A scoping review

**DOI:** 10.5281/zenodo.13934643

**Published:** 2024-10-15

**Authors:** Ifeoma C. Ezenyi, Kim Picozzi, John I. Amaka, Obi P. Adigwe

**Affiliations:** 1 Department of Pharmacology and Toxicology, National Institute for Pharmaceutical Research and Development, Federal Ministry of Health, Abuja, Nigeria.; 2 Deanery of Biomedical Sciences, College of Medicine and Veterinary Medicine, The University of Edinburgh, 1 George Square, EH8 9JZ Edinburgh, United Kingdom.; 3 Center for Tropical and Emerging Global Diseases, College of Veterinary Medicine, University of Georgia, Athens, USA.; 4 Office of the Director-General/Chief Executive Officer, National Institute for Pharmaceutical Research and Development, Federal Ministry of Health, Abuja, Nigeria.

## Abstract

**Background:**

Malaria is a leading cause of mortality in children aged 5 years and below in Nigeria. Treatment guidelines stipulate among other recommendations, testing by microscopy or a rapid diagnostic test (RDT) before treatment. Non-adherence to these guidelines portends a challenge, especially among vulnerable under-five children. This study explored the factors influencing Nigerian public health workers’ (HWs) adherence to these guidelines in under-five children.

**Methods:**

A review of literature published between 2011- 2023 was conducted on Web of Science, Ovid Embase, Medline, Global Health, CAB Abstracts, Scopus, and Global Index Medicus. Data was extracted and analyzed under 4 themes: diagnosis, compliance with test results, use of recommended treatment, post-treatment counselling and severe malaria management.

**Results:**

Nineteen (19) studies were included for review. Training and supervision, RDT and antimalarial availability, good knowledge of, and positive perception of RDTs promoted adherence to mRDT use. A lack of confidence in RDTs and age (≥ 40 years) fuelled presumptive treatment, especially among clinicians. mRDT and artemisinin-based combination therapy (ACT) stockouts dissuaded HWs from adhering to case management guidelines. Caregiver pressure for treatment was identified as a barrier to compliance with test results.

**Conclusions:**

It is important to design context-specific strategies to improve adherence to guidelines for malaria case management, especially in under-five children. Training on the guidelines should be tailored, needs-based, and continuous, and HWs should be supportively supervised in implementing case management. Maintaining an adequate supply of quality-assured mRDTs and antimalarials can facilitate adherence to the guidelines.

## Introduction

Malaria remains a serious health challenge in sub-Saharan Africa. Children below the age of five and pregnant women are particularly susceptible to severe malaria and can die from the disease, especially in high-transmission areas [1]. An estimated 249 million malaria cases occurred globally in 2022, up from 244 million cases in 2021 [2,3]. The African continent bears the highest burden, with Nigeria alone accounting for about 27% of the global malaria cases [3].

The Global Technical Strategy for malaria targets a reduction in malaria mortality rate and malaria case incidence by at least 75% by 2025 [4]. Nigeria has also set targets to reduce parasite prevalence to < 10%, and to reduce malaria-attributable mortality to < 50 deaths per 1,000 live births by 2025 [5]. Presently, malaria prevalence in children aged 6 – 59 months in Nigeria is 22% and this is higher than the set target [6]. Given the contribution of this population to the malaria burden in Nigeria, it is important to identify bottlenecks to malaria management in this group.

In 2010, the World Health Organization (WHO) recommended that any suspicion of malaria in a patient should be confirmed by prompt parasitological testing using malaria rapid diagnostic tests (mRDTs) or microscopy before treatment, except where testing cannot be accessed within 2 hours of a patient presenting for treatment [7]. Following this, the WHO launched a ‘test, treat, track’ (T3) approach for malaria case management in 2012 [8]. Soon after, several countries adopted these recommendations and have continued to implement the guidelines. Nigeria has also integrated this recommendation into its national guidelines for the treatment of malaria [9].

Adherence to national guidelines by healthcare providers is essential for harmonising malaria case management and achieving success of the policy, which is also vital for reducing the malaria burden in the country. Generally, training and supervision, antimalarial commodity supply, and availability were system-level factors affecting adherence. At the health worker (HW) level, good knowledge and perception about mRDTs, confidence in test results, and HW qualification/cadre influenced adherence. These findings were documented from other studies in public health facilities in Ghana, Kenya, Malawi, and Uganda [10-14]. The health systems in these malaria-endemic countries are like that of Nigeria, in terms of a cascade of care from primary to tertiary levels, and adoption of WHO-recommended guidelines for malaria case management.

Given that nearly a quarter of Nigeria’s under-five children population harbour malaria parasites and can potentially suffer from fatal disease, it is pertinent to assess the factors that influence the compliance of HWs with national guidelines for the treatment of malaria in under-five children. This scoping review aims to identify the barriers and facilitators for adherence of public health workers to these guidelines in managing under-five children with suspected malaria.

## Methods

A five-step scoping review method was adopted, as outlined by Arksey and O’Malley [15]. This involves 5 steps, which include: (i) setting the research question, (ii) identifying relevant studies, (iii) selecting studies, (iv) charting and synthesizing data (iv) collating, summarizing, and reporting the results. This study also followed other guidance for conducting scoping reviews and is aligned with the Preferred Reporting Items for Systematic Reviews and Meta-Analyses extension for Scoping Reviews (PRISMA-ScR) [16,17]. This is presented in Appendix 1.

### Research question

This scoping review was guided by the research question, “What are the factors associated with health worker adherence, or lack thereof, to national guidelines for malaria diagnosis and treatment among under-five children in Nigeria?”

### Identification of relevant studies

#### Search strategy

A search strategy was developed in collaboration with a staff of the University of Edinburgh Library. A title and abstract search from PubMed advanced search builder was carried out to identify ‘indicator’ papers. PubMed IDs (PMIDs) of relevant indicator publications identified were entered in the Yale MeSH analyzer tool (https://mesh.med.yale.edu/) to obtain subject headings and keywords. This was followed by an expanded, secondary literature search on Web of Science, Ovid (Embase, Medline, Global Health, CAB Abstracts), Scopus, and Global Index Medicus. Additional studies were identified using a backward and forward snowballing approach [18]. The literature search was performed from March to April 2023, results of three database search strategies are shown in Appendix 2. Search results were imported into Covidence data management software, to remove duplicate records. Further abstract and article review was done following inclusion and exclusion criteria to determine eligibility.

#### Inclusion and exclusion criteria

Papers published in English language between 2011 – 2023 were included, as Nigeria adopted the global policy on malaria case management in 2011. Studies conducted at the community level, or in primary, secondary, or tertiary health settings assessing compliance to guidelines among public HWs were included. Studies assessing adherence in the private (for-profit) sector or among caregivers of children alone, or whose data on the public health worker was not disaggregated were not included in screening. Multi-country studies which involved Nigeria were included. Studies that did not specify under-fives as the patient population, but assessed health care providers at primary care levels or community health workers were deemed eligible for inclusion. Reviews, retrospective analyses of health facility records, and case reports were excluded.

### Data charting and synthesis

A charting template, shown in Table 1, was used to record relevant information from included publications [19].

**Table 1.** Template for data extraction.

S/No.	Variable	Description
1	Authors, year	Names of authors on the publication and Year of publication
2	Title	Title of publication
3	Aim	The aims and objectives that informed the conduct of the study
4	Study design and data collection method	Type of study design adopted and data collection instrument
5	Participants	Type and number of Participants in the study
6	Key findings	Information obtained from the study based on the review research question

## Results

**Figure 1.**
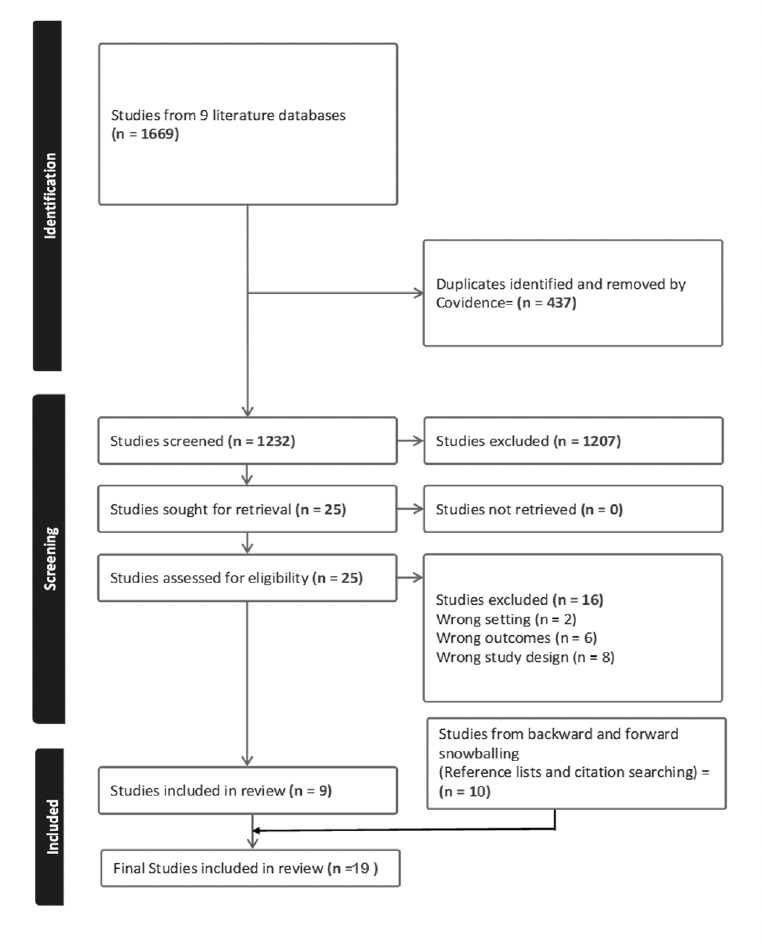
PRISMA flow chart showing the process study selection.

**Figure 2.**
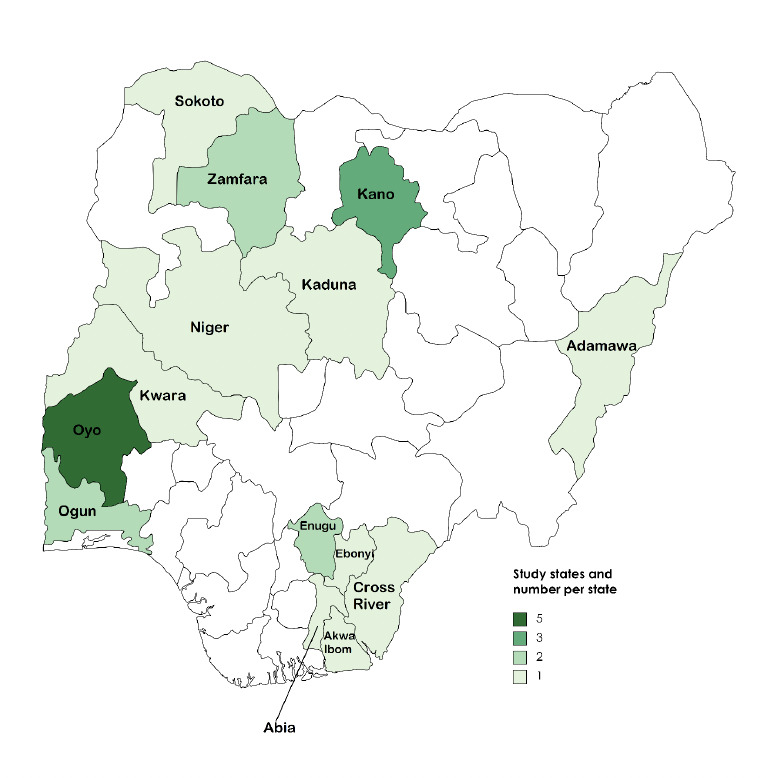
Map of Nigeria showing states where selected studies were conducted.

The primary and secondary database searches yielded 9 eligible studies (Figure 1). Further forward and backward reference and citation searches identified an additional 10 studies that were eligible for inclusion. An increasing trend was observed in the number of reports from 2012 – 2020. Only one eligible publication was obtained for the immediate period (2012 – 2014) following the release of the national guidelines in 2011, followed by 4 studies between 2015-2017. The number of publications reached a maximum between 2018 – 2020 (n=8), but subsequent years (2021-2023) witnessed a decline in studies with similar themes (n=3). A map of Nigeria showing the 14 states where the studies were conducted is shown in Figure 2. Most of the studies were conducted in Oyo state in the southwest of the country. There were 3 multi-state studies; one study was done in five states, while 2 studies were carried out in 2 states each. Two studies were not shown because the participants were visiting the state and not practicing in the state where the surveys were done. Three studies were based on malaria-endemic, multi-country projects (3 countries per study) which included Nigeria. The most studied healthcare providers were community health workers (CHWs) in primary healthcare settings, other HWs studied were nurses and clinicians; overall, the sample size of study participants ranged from 8 – 325 (Appendix 3).

For data extraction and analysis, 4 domains of adherence to the national guidelines emerged as themes for data analysis: (i) Use of malaria rapid diagnostic tests or microscopy (ii) Compliance with test results (iii) Use of recommended treatment for malaria (uncomplicated or severe) (iv) Referral practices and post-treatment services after severe malaria management. Some studies included for review contributed to more than one theme.

### Malaria diagnosis using mRDTs or microscopy

Health worker (HW) training facilitated the use of microscopy or mRDTs among female CHWs in Oyo state, Nigeria [20]. Additionally, direct observation of CHWs contributed to their proficiency. However, the study's applicability was limited by the exclusion of ten CHWs unable to perform RDTs and keep records according to the training protocol, as well as the absence of five CHWs during supervisory visits [20]. In contrast, another study among health workers in Niger state reported that cascade training and supportive supervision implementation did not enhance mRDT utilisation, despite the provision of free testing services [21]. The low adherence was attributed to context-specific factors, especially mRDT stock-outs [21].

Similar to this was the finding from a study conducted in 6 states which reported that mRDT stockout negatively affected adherence to mRDT use [22]. The study revealed good mRDT knowledge and acceptance among health facility staff, but the use of mRDT varied across states, with the lowest usage in Lagos (30.2%) and Ogun (9%) states, while HWs in Enugu completely abstained from mRDT use due to perceived sensitivity and accuracy issues [22]. Other factors besides the unavailability of mRDTs which hindered adherence to malaria testing in Enugu state were delayed microscopy results, and heavy workload [23]. Presumptive treatment of malaria was common among doctors and laboratory scientists due to concerns about delays, caregiver demands, and the absence of mRDTs in consulting rooms [23].

A study among HWs in Zamfara state indicated that nurses/midwives and laboratory scientists/technicians were more likely to use mRDTs compared to doctors, community health extension workers, and community health officers [27]. Good knowledge of mRDT, trust in mRDT results, previous training in malaria case management, and free mRDT services in facilities were also strong predictors of mRDT use among the HWs [27]. Age and income level of HWs were also found to influence adherence to malaria diagnosis in the continuum of care for febrile children [28, 29]. Younger HWs were more likely than older ones to adopt good practices relating to malaria diagnosis. Low-income HWs earning below the minimum wage of ₦30,000 were less likely to adopt good testing practices [29].

### Compliance with test results

The impact of training on HW compliance with test results showed mixed outcomes in in the studies analysed [20,21,26]. In one study, CHW training was linked with complete compliance with diagnostic test results [20]. Similarly, HW training and supervisory visits decreased the prescription of artemisinin-based combination therapy (ACTs) for mRDT-negative patients in Kano and Zamfara states during a 3-year intervention period [26]. In contrast, the implementation of cascade training and direct supervision of HWs in Niger state did not result in better compliance with test results [21]. Interestingly, the non-intervention group largely outperformed the intervention group in appropriately treating mRDT-negative children (93% versus 37%) [21]. This discrepancy could have been driven by a lack of confidence in mRDT-negative results among the intervention group, or as highlighted by the authors, the inability of cascade training to reach the target group [21]. This highlights the need for training to be context-tailored and where possible, complemented with supervision to improve compliance to test results.

Another intervention study across three countries investigated the factors influencing the prescription of ACTs for mRDT-negative children among trained CHWs [30]. The intervention, primarily training, was deemed effective but a small percentage of febrile, mRDT-negative children in Nigeria received an ACT from the trained CHWs [30]. Further microscopy and quality assurance tests revealed that a significant portion of mRDT-negative cases were positive for malaria. This highlights the potential risk of withholding ACTs from febrile children with malaria, based on a false negative mRDT result.

The qualification of HWs and pressure from caregivers for treatment influence compliance with malaria test results. One study in primary and secondary health facilities in Sokoto state, Nigeria reported that compliance was more likely among junior community health extension worker certificate holders and diploma holders, than other higher degree holders [31]. In another study, factors such as caregiver educational status and age of the child (5 years and below) were associated with ACT prescription for mRDT-negative patients [22]. The HWs prescribed ACTs due to pressure from caregivers who expressed expectations of receiving antimalarials since they were freely provided by the government. Similarly, a barrier to compliance with test results among CHWs in Oyo state was the failure of caregivers to accept that RDT-negative children should not be treated with an antimalarial [32].

A poor perception of RDTs among health workers was identified as a significant factor influencing the prescription of ACTs to RDT-negative patients in a survey conducted in Ebonyi state, southeast Nigeria [33]. Compliance with RDT results was better among primary healthcare workers than those in higher health facilities. In a few studies, having good knowledge and a positive perception of mRDTs were correlated with adherence to test results and vice versa [25, 26]. One survey in Oyo state, Nigeria, revealed that 26.0% of HWs surveyed prescribed antimalarials to mRDT-negative patients, reportedly due to a lack of training and poor knowledge of the causes of fever [34].

### Use of recommended treatment for uncomplicated and severe malaria

The availability of recommended medicine and training can considerably influence the use of appropriate treatment but evidence showed that these may not always correlate with good prescription practices. Trained female CHWs, under direct supervision, effectively adhered to treatment protocols, administering the correct dose of ACTs or rectal artesunate in children [20]. The availability of ACTs facilitated good prescription practices among health workers in Ogun state, with the high availability of antimalarials in public health facilities being the main driver behind the prescription of recommended drugs [35]. Supporting this was the finding that inconsistencies in ACT supply led to the prescription of artesunate monotherapy in some rural health facilities [21].

Factors such as training on malaria case management, access to national guidelines, and availability of malaria diagnostic tools may not facilitate the use of recommended treatment in some contexts. Notwithstanding the high availability of ACTs in hospitals in Kano state, compliance with ACT use was low, with only 4% of admitted non-severe test-positive cases being treated with an ACT, while artemether monotherapy was prevalent even among test-negative patients [36]. Further, despite high levels of knowledge among health workers regarding testing and treatment for severe malaria, there were knowledge gaps regarding artesunate dosing recommendations, particularly for children weighing less than 20 kg [36]. This was also reported from a survey among pediatricians attending an update course in Edo state, which revealed inadequate knowledge of correct prescription practices for uncomplicated and severe malaria, highlighting the importance of formal training in guideline-based management [37].

The acceptability of rectal artesunate for severe malaria was an indicator of its pre-referral use by HWs. In a study assessing the acceptability of pre-referral rectal artesunate (RAS) among health workers, high perception and acceptance of RAS for severe malaria in children were reported among Nigerian CHWs and primary healthcare workers [38]. Positive perception and high acceptability may serve as motivating factors for RAS uptake, especially when stock is available.

### Referral practices and post-treatment services after severe malaria management

At the primary healthcare level in communities, malaria-positive cases with symptoms suggestive of disease severity should be promptly referred to higher-level health facilities for adequate management. Post-treatment services cover caregiver counseling on how to use oral medication after parenteral treatment for severe malaria. An examination of the data extracted from all the selected studies showed that adherence to guidelines for severe malaria management was the least studied domain concerning malaria case management. However, the refusal of caregivers to comply with referral advice offered by CHWs was identified as a barrier to this service [32]. In a study among trained CHWs, adherence was low for post-treatment counseling of home-based caregivers [20]. Also, insufficient knowledge of ACT doses in children among some physicians and nurses was attributed to poor adherence to post-severe malaria management [36,37].

## Discussion

Understanding and addressing the factors influencing adherence to malaria case management in under-five children by Nigerian health workers are important for enhancing malaria management and reducing the childhood malaria burden and its impact. The main factors identified include training, mRDT and ACT availability, health worker qualification and years of experience, perception of mRDTs for malaria diagnosis, knowledge of recommended treatment and their doses, and caregiver pressure for treatment. The finding that adherence to malaria case management guidelines was higher at primary healthcare levels and among lower-level professional cadres than higher-level HWs is supported by similar reports among HWs in Ghana and Uganda [10,39]. Nursing aides and nurses showed higher adherence compared to clinicians and adherence was associated with knowledge of the correct dosage of antimalarials, including parenteral artesunate [10]. Low adherence was also noted among doctors in Ghana who demonstrated limited knowledge of appropriate prescriptions for uncomplicated malaria [39].

Implementing tailored interventions at the facility level to improve knowledge, perceptions, and practices regarding malaria diagnosis and treatment can improve adherence to case management guidelines. Given the variability in training outcomes across different types of health facilities (primary, secondary, or tertiary) and HW cadres observed in this review, it is necessary to identify training needs such that any training implemented will be needs-based and fit for purpose. Additionally, a well-coordinated approach involving all relevant stakeholders is essential to improve training effectiveness. An effective training strategy should be acceptable, accessible, feasible, and scalable. Standardised training modules covering all aspects of diagnosis and treatment can facilitate certified training for various HW cadres. Highly trained clinicians may have higher workloads and may not always devote time to participate in training programs. Refresher courses integrated into periodic update programs and continuing education initiatives for paediatricians and family medicine physicians can help ensure clinicians stay updated on national guidelines. Additionally, visual job aids can reinforce knowledge of correct dosing schedules for parenteral antimalarials, as demonstrated by Machini et al. [40].

Mobile learning interventions and supportive packages, community monitoring, and problem-solving approaches have shown promise in improving service delivery quality, particularly in remote settings [41,42]. A systematic review highlighted how the integration of digital technologies during supervision enhanced HW performance and the use of health system information and key performance data for prioritising low-performing areas during supervisory visits [43]. This strategy holds the potential for improving the proficiency of a trained healthcare workforce and providing tailored solutions for increasing adherence to case management guidelines.

Testing febrile cases before treatment is a crucial practice that can preserve the effectiveness of ACTs and slow down the emergence of drug resistance. It can also improve the management of non-malarious febrile illnesses in children, when they test negative for malaria. Nigeria initiated the rollout of mRDTs in a phased program at primary healthcare (PHC) facilities starting in 2011 [22]. According to the national malaria diagnosis and treatment guidelines, children under five with suspected malaria should only receive empirical treatment when diagnostic tests are unavailable [9]. This scenario is common in many health facilities where mRDT supply is erratic or non-existent, and infrastructure for microscopic diagnosis is lacking. While microscopy can be more sensitive and accurate than mRDTs, relying solely on microscopy for malaria diagnosis is often impractical due to a shortage of quality-assured malaria microscopy. Thus, mRDT use is an important pillar for malaria diagnosis in Nigeria. Therefore, it is imperative to train and motivate HWs to use mRDTs.

Previous research has shown that a negative perception of mRDTs is predictive of non-compliance with test-negative results [44,45]. To instil trust in mRDTs among HWs, enhancing the quality assurance system for mRDTs is essential. Ensuring the supply and proper storage of quality-assured RDTs are crucial steps in building confidence in their use and promoting the adoption of RDTs. Typically, mRDTs can be maintained at room temperature of 1- 45ºC and have a shelf life of 24-30 months [46]. Thus, quality can be affected by various factors, including high temperatures and humidity. Poor quality mRDTs can compromise result reliability, underscoring the importance of closely monitoring supply chains and mRDT storage conditions to maintain their quality. Their transport, storage, and handling should follow WHO guidance on handling of temperature-sensitive products [46].

False negative mRDT results pose challenges in malaria diagnosis, potentially leading to the withholding of ACTs for febrile patients, thereby increasing the risk of severe malaria complications. The detection of a malaria antigen, histidine-rich protein, in infected blood forms the basis of most mRDTs. However, the absence of histidine-rich protein 2 (hrp2) and histidine-rich protein 3 (hrp3) genes in certain *Plasmodium falciparum* strains may contribute to false-negative results with some RDTs. Studies have reported the presence of P. falciparum strains lacking these proteins in malaria-endemic regions across Asia, Central and South America, and Africa [47,48]. While Nigeria predominantly employs hrp2-based mRDTs, recent studies have documented Pfhrp2 and Pfhrp3 deletions in the country [48,49]. Nationwide studies are warranted to ascertain the prevalence of Pfhrp2 and Pfhrp3 deletions, which can inform policy decisions regarding the type of mRDTs to procure and utilise domestically. The sensitivity of mRDTs is influenced by the parasite density in blood samples, with very low parasite densities potentially going undetected by standard mRDTs [50]. New ultra-sensitive malaria tests have been developed to detect genetic traces of *Plasmodium* parasites with greater sensitivity than standard mRDTs [51]. However, operational research is needed to determine their clinical superiority over standard mRDTs for primary care settings, especially concerning the diagnosis and treatment of children. A 2019 study showed that mRDT diagnostic accuracy was 79% in children aged 0 – 59 months, with weaker predictive accuracy compared to microscopy [52]. Another study reported that mRDT diagnostic accuracy was 64.1% among febrile under five children [53]. Based on these, there may be a need to support mRDT testing with microscopy where possible, for better diagnostic outcomes. Better malaria diagnostic sensitivity has been observed when mRDTs were combined with microscopy in diagnosing malaria [54].

Health worker prescribing patterns for malaria are often contingent upon the availability of medications at health facilities. Therefore, regular monitoring of stock availability is crucial to ensure the accessibility of ACTs while discouraging the supply of oral monotherapies to health facilities. To effectively implement national malaria case management guidelines, the Federal Ministry of Health, through the National Malaria Elimination Programme and non-government partners, should enhance the supply of malaria test kits and ACTs. Government and political stakeholders' involvement in filling funding gaps for antimalarial commodity procurement is essential. Sub-national reporting on stock and inventory management must be diligently executed to prevent stock-outs and product expiration. Improving inventory tracking tools and providing training to health workers on their use can facilitate the prediction of procurement and supply needs based on usage patterns and malaria seasonality. A notable example involves the use of call centers to collect monthly data on stock availability, leading to a reduction in stockout rates compared to traditional paper-based systems [55]. This low-tech approach can be adapted to complement existing inventory management systems in Nigeria.

Evidence on factors influencing health worker compliance with pre- and post-referral recommendations in Nigeria is limited, highlighting the need for further research. Adherence to referral instructions and post-referral treatment recommendations is critical for ensuring favourable outcomes in severe malaria case management. In severe malaria cases, community-delivered pre-referral management should be followed by referral to higher-level health facilities.

### Recommendations

To promote adherence to malaria case management guidelines, a multi-faceted approach should be adopted. There is a need to increase awareness of the guidelines, especially for test-based diagnosis of malaria and compliance with test results. It is also important to train health workers on the continuum of care for febrile under-five children, and malaria case management guidelines. Training and supportive supervision should be tailored to the context. Staff roles and age, type of training, implementation strategies, and supervision needs should be considered when designing training programs. It is paramount that the supply of quality-assured mRDTs, ACTs, and other treatments for severe malaria is guaranteed, and that stock availability of these commodities in health facilities remains adequate.

### Limitations of the study

One reviewer conducted data charting and extraction. Most studies included in this review used quantitative data collection tools (questionnaires) and only a few collected qualitative data. This constitutes another limitation as data obtained using questionnaires can be subjective when self-reported, and is often not a complete representation of the target population response, especially when the sample size is small compared to the general target population. Also, questionnaire response rates hardly reach 100%, which may give an incomplete representation of the study population. Participant responses are usually more restricted in questionnaires compared to information gathered by qualitative methods. Lastly, an electronic search of databases that was adopted may miss some relevant papers on a subject. The process is not 100% efficient as keyword and subject headings used in the search strategy may omit studies that may have been categorised under different keywords and headings.

## Conclusions

This review was employed to compile evidence of factors associated with adherence to malaria case management guidelines in under-five children among public health care providers in Nigeria. The factors were categorised by themes around the use of diagnostic tests, test result compliance, use of recommended treatment, and pre- and post-referral practices for children with severe malaria. The findings reveal that health worker knowledge about the guidelines alone does not always suffice for guideline adherence. Income level, category of health worker (clinician, nurse, community health worker), and age were individual-level predictors of adherence. Institutional factors which can be addressed include bolstering health worker training and supervision, and developing a mechanism to guarantee a regular supply of quality-assured malaria case management commodities for successful implementation of the country’s National Malaria Policy. This is vital for reducing the malaria burden in the country and ultimately contributes to the attainment of the 2030 global malaria targets.

## Appendix 1. Preferred Reporting Items for Systematic reviews and Meta-Analyses extension for Scoping Reviews (PRISMA-ScR) checklist [16].

**Table:**

Section	Item	Prisma-scr checklist item	Reported on page #
**Title**	1	Identify the report as a scoping review.	1
**Abstract**			
Structured summary	2	Provide a structured summary that includes (as applicable) background, objectives, eligibility criteria, sources of evidence, charting methods, results, and conclusions that relate to the review questions and objectives.	1
**Introduction**			
Rationale	3	Describe the rationale for the review in the context of what is already known. Explain why the review questions/objectives lend themselves to a scoping review approach.	2
Objectives	4	Provide an explicit statement of the questions and objectives being addressed regarding their key elements (e.g., population or participants, concepts, and context) or other relevant key elements used to conceptualize the review questions and/or objectives.	2
**Methods**			
Protocol and registration	5	Indicate whether a review protocol exists; state if and where it can be accessed (e.g., a Web address); and if available, provide registration information, including the registration number.	Available upon request
Eligibility criteria	6	Specify characteristics of the sources of evidence used as eligibility criteria (e.g., years considered, language, and publication status), and provide a rationale.	2
Information sources	7	Describe all information sources in the search (e.g., databases with dates of coverage and contact with authors to identify additional sources), as well as the date the most recent search was executed.	2
Search	8	Present the full electronic search strategy for at least one database, including any limits used, such that it could be repeated.	Appendix 2
Selection of sources of evidence	9	State the process for selecting sources of evidence (i.e., screening and eligibility) included in the scoping review.	2
Data charting process	10	Describe the methods of charting data from the included sources of evidence (e.g., calibrated forms or forms that have been tested by the team before their use, and whether data charting was done independently or in duplicate) and any processes for obtaining and confirming data from investigators.	3
Data items	11	List and define all variables for which data were sought and any assumptions and simplifications made.	Table 1
Critical appraisal of individual sources of evidence	12	If done, provide a rationale for conducting a critical appraisal of included sources of evidence; describe the methods used and how this information was used in any data synthesis (if appropriate).	N/A
Summary measures	13	Not applicable for scoping reviews.	N/A
Synthesis of results	14	Describe the methods of handling and summarizing the data that were charted.	3
Risk of bias across studies	15	Not applicable for scoping reviews.	N/A
Additional analyses	16	Not applicable for scoping reviews.	N/A
**Results**			
Selection of sources of evidence	17	Give numbers of sources of evidence screened, assessed for eligibility, and included in the review, with reasons for exclusions at each stage, ideally using a flow diagram.	3
Characteristics of sources of evidence	18	For each source of evidence, present characteristics for which data were charted and provide the citations.	Appendix 3
Critical appraisal within sources of evidence	19	If done, present data on critical appraisal of included sources of evidence (see item 12).	N/A
Results of individual sources of evidence	20	For each included source of evidence, present the relevant data that were charted that relate to the review questions and objectives.	Appendix 3
Synthesis of results	21	Summarize and/or present the charting results as they relate to the review questions and objectives.	3-6, Appendix 3
Risk of bias across studies	22	Not applicable for scoping reviews.	N/A
Additional analyses	23	Not applicable for scoping reviews.	N/A
**Discussion**			
Summary of evidence	24	Summarize the main results (including an overview of concepts, themes, and types of evidence available), link to the review questions and objectives, and consider the relevance to key groups.	6-8
Limitations	25	Discuss the limitations of the scoping review process.	8
Conclusions	26	Provide a general interpretation of the results with respect to the review questions and objectives, as well as potential implications and/or next steps.	8
**Funding**	27	Describe sources of funding for the included sources of evidence, as well as sources of funding for the scoping review. Describe the role of the funders of the scoping review.	8

## Appendix 2. Electronic search strategy.

**Table:**

EMBASE		
#	Search query	Search (31 Mar results 2023)
1	exp malaria falciparum/ or exp cerebral malaria/ or exp malaria/	98,430
2	exp health care personnel/	1,911,075
3	(nurse* or doctor* or physician* or "health worker*").mp. [mp=title, abstract, heading word, drug trade name, original title, device manufacturer, drug manufacturer, device trade name, keyword heading word, floating subheading word, candidate term word]	1,408,124
4	2 or 3	2,551,847
5	exp protocol compliance/ or health personnel attitude/	103,540
6	(compliance or adhere*).mp. [mp=title, abstract, heading word, drug trade name, original title, device manufacturer, drug manufacturer, device trade name, keyword heading word, floating subheading word, candidate term word]	660,746
7	5 or 6	741,664
8	antimalarial agent/ or arteether/ or artemether/ or artemisinin/ or artesunate/	39,000
9	exp case management/	13,516
10	(ACT or treat* or prescri*).mp. [mp=title, abstract, heading word, drug trade name, original title, device manufacturer, drug manufacturer, device trade name, keyword heading word, floating subheading word, candidate term word] exp malaria rapid test/ or diagnos*.mp. [mp=title, abstract, heading word, drug trade name,	10,331,149
11	original title, device manufacturer, drug manufacturer, device trade name, keyword heading word, floating subheading word, candidate term word]	7,148,271
12	exp patient referral/	151,188
13	8 or 9 or 10 or 11 or 12	14,972,752
14	exp child/ or infant/ or preschool child/ or toddler/	2,900,374
15	("under five*" or u5).mp. [mp=title, abstract, heading word, drug trade name, original title, device manufacturer, drug manufacturer, device trade name, keyword heading word, floating subheading word, candidate term word]	11,696
16	14 or 15	2,905,000
17	1 and 4 and 7 and 13 and 16	245
18	limit 17 to yr="2011 - 2023"	193

**WEB OF SCIENCE**		
**#**	**Search query**	**Search (5 Apr results 2023)**
1	ALL=(malaria OR falciparum OR “cerebral malaria” OR “severe malaria”)	131,663
2	ALL=(compliance OR adhere* OR practice* OR attitude*)	3,360,593
3	ALL=(“health worker” OR doctor* OR “health personnel” OR nurse* OR physician*)	1,438,013
4	ALL=(“case manage*” OR guideline* OR test* OR rdt OR microscop* OR diagnos* OR act OR antimalarial* or artesunate OR artemisinin* OR prescri* OR refer*)	15,342,581
5	ALL=(child* OR infant* OR “under five*” OR preschool)	3,736,459
6	#1 AND #2 AND #3 AND #4 AND #5	259
7	#1 AND #2 AND #3 AND #4 AND #5Timespan: 2011-01-01 to 2023-12-31	195

**SCOPUS**		
**#**	**Search query**	**Search (5 Apr results 2023)**
1	TITLE-ABS-KEY ( malaria OR falciparum OR "cerebral malaria" OR "severe malaria" )	390,902
2	TITLE-ABS-KEY ( compliance OR adhere* OR practice* OR attitude* )	13,912,212
3	TITLE-ABS-KEY ( "health worker" OR doctor* OR "health personnel" OR nurse* OR physician* )	4,211,620
4	TITLE-ABS-KEY ( "case manage*" OR guideline* OR test* OR rdt OR microscop* OR diagnos* OR act OR antimalarial* ORartesunate OR artemisinin* OR prescri* OR refer* )	36,539,103
5	TITLE-ABS-KEY ( child* OR infant* OR "under five*" OR preschool )	9,387,840
6	1 AND 2 AND 3 AND 4 AND 5	14,314
7	TITLE-ABS-KEY (1 AND 2 AND 3 AND 4 AND 5) AND PUBYEAR > 2010 AND PUBYEAR < 2024	423

## Appendix 3. Characteristics of studies included in the scoping review.

**Table:**

Reference	Study title	Aim	Study design & Data collection method	Participants	Key findings
[20]	The fidelity of implementation of recommended care for children with malaria by community health workers in Nigeria	To assess the feasibility, acceptability, and cost-effectiveness of a package for the diagnosis and treatment of uncomplicated and severe malaria in children under 5 years of age delivered at the community level	Quantitative, Hospital-based observation with a standard checklist	35 female CHWs	Training improved CHW adherence to malaria treatment guidelines (use of RDTs and compliance with results), especially among those with healthcare-related occupations. However, adherence was lowest for post-treatment initiation counseling of home-based caregivers
[21]	Evaluation of a capacity building intervention on malaria treatment for under-fives in rural health facilities in Niger State, Nigeria	To assess the effectiveness of the programme on malaria diagnosis and prescription practices among febrile under-fives attending rural health facilities in Niger state	Mixed method, Questionnaire-based survey, and direct observation	171 HWs across 28 health facilities	Within a context of significant ACT and RDT stockouts, an intervention including cascade training and on-the-job supervision did not improve appropriate management of under- fives compared to non-intervention comparison facilities
[22]	Provider and patient perceptions of malaria rapid diagnostic test use in Nigeria: a cross-sectional evaluation	To evaluate provider and patient perceptions on the use of RDT in malaria case management at the primary healthcare level in Nigeria	Mixed method, Questionnaire-based survey Health facility staff interviews	120 health facility staff in 120 health centers	Confidence in mRDT results favored compliance with test results, whereas some HWs do not use RDTs due to the perceived inaccuracy of mRDT results
[23]	Qualitative study of presumptive treatment of childhood malaria in third tier tertiary hospitals in southeast Nigeria: a focus group and in-depth study	To determine the perspectives of patients, health-care providers and laboratory scientists regarding the confirmation of malaria in a tertiary hospital	Qualitative, in-depth interviews	8 doctors and 2 laboratory scientists	Among doctors, presumptive treatment without testing is driven by malaria endemicity, parental pressure, mRDT unavailability in clinic, long wait times for laboratory confirmation, and false negative mRDT results. Lab scientists’ capacity does not match the high test request load, and malaria test requests are not seen as deserving of urgent attention
[24]	Malaria Diagnostic Testing among Public Health Physicians in Nigeria	To determine the use of malaria diagnostic test by Public Health Physicians	Quantitative, self- administered questionnaire- based survey	125 clinicians practicing in different public health facilities	Physicians ≥40 years old were more likely to rely on clinical diagnosis than laboratory testing in the last child case seen
[25]	Compliance with Malaria Rapid Diagnostic Test Results and Correlates among Clinicians in Uyo, Akwa Ibom State, Nigeria: 2018	To determine compliance with malaria RDT results and the associated factors among clinicians in Uyo, Akwa Ibom State	Quantitative, self- administered semi-structured questionnaire	170 clinicians in a public health facility	Compliance with RDT negative and positive results was higher among clinicians with good knowledge of RDT use and positive perception of RDT usefulness
[26]	Malaria Frontline Project: strategic approaches to improve malaria control program leveraging experiences from Kano and Zamfara States, Nigeria, 2016–2019	To describe the project implementation and its contributions to malaria control programs in health facilities in Kano and Zamfara States, Nigeria	Mixed methods, Health worker questionnaire-based survey, client exit interviews and supervisory visit reports	338 HWs in 46 PHCs and 45 General Hospitals	The proportion of health workers who adhered to national guidelines for malaria diagnosis and treatment increased following the implementation of the use of tailored training materials, job aids, supportive supervision
[27]	Predictors of malaria Rapid Diagnostic Tests’ utilisation among healthcare workers in Zamfara State	To investigate the knowledge of mRDT, mRDT availability and use as well as factors influencing mRDT utilizAation in health facilities in Zamfara State	Quantitative, Questionnaire- based survey	306 HWs in 54 health facilities	Nurses and midwives were more likely to adhere to mRDT use, compared to CHWs. Predictors of mRDT use were good knowledge of mRDT, trust in mRDT results, prior training on mRDT, and availability of free mRDTs
[28]	Knowledge, use of mRDT, and malaria control practice among health care workers in three Local Government areas of Kano state, North-Western-Nigeria	To investigate mRDT knowledge and use, and malaria control practice by healthcare workers in 3 Local government areas (LGAs) of Kano State, Nigeria	Quantitative, self- administered Questionnaire	263 HWs in 80 primary health facilities	Good mRDT knowledge was a predictor of mRDT use
[29]	Assessing the knowledge and practices of primary healthcare workers on malaria diagnosis and related challenges in view of COVID-19 outbreak in a Nigerian Southwestern metropolis	To determine the ability of PHC HWs to correctly diagnose malaria and differentiate between malaria and COVID-19 infections in selected primary healthcare facilities	Quantitative, electronic Questionnaire	261 HWs in primary health centers	Poor practices were attributed to the unavailability of mRDTs, inadequate training, and the absence of continuous capacity improvement. Low income also posed a barrier to testing adherence
[30]	Compliance With Malaria Rapid Diagnostic Testing by Community Health Workers in 3 Malaria- Endemic Countries of Sub-Saharan Africa: An Observational Study	To quantify the number of patients who were prescribed and treated with ACTs when the RDT was negative, and to understand the reasons and risk factors relating to this behavior	Qualitative, Structured interviews	42 CHWs	A lack of adherence to mRDT test results is relatively uncommon when CHWs are trained and well-supervised. However, more than half of the Nigerian CHWs who administered ACTs to mRDT-negative children did so because of insistence by the child’s caregiver
[31]	Adherence to malaria rapid diagnostic test result among healthcare workers in Sokoto metropolis, Nigeria	To determine HWs adherence to RDT results and factors influencing their adherence to test results	Quantitative, Questionnaire- based survey	262 HWs in primary and secondary health facilities	Predictors of adherence to test results were the presence of fever in the patient, expectation from the patient of HW to give an antimalarial, and training on malaria case management
[32]	Motivation of Community Health Workers in Diagnosing, Treating, and Referring Sick Young Children in a Multicountry Study	to explore CHWs' motivation and inspiration to work for their communities.	Qualitative, focus group discussion	25 CHWs in 8 primary health centers	Low level of motivation and general level of satisfaction as a CHW may affect diagnosing, treating, and referring practices. Frequent stockouts of drugs and supplies, the volume of work, refusal of caregivers to comply with treatment/referral advice, or to accept that the child should not be treated when the rapid diagnostic test (RDT) is negative were reported as barriers
[33]	Health workers’ perception of malaria rapid diagnostic test and factors influencing compliance with test results in Ebonyi state, Nigeria	To assess HWs perception of mRDT and factors that influence ACT prescription to patients with negative test results in Ebonyi state Nigeria	Quantitative, interviewer- administered open data kit (ODK) based semi-structured questionnaire	220 HWs in 37 public health facilities	HWs’ poor perception of mRDTs promoted the prescription of ACT to patients with negative mRDT results
[34]	Practice of antimalarial prescriptions to patients with negative rapid test results and associated factors among health workers in Oyo State, Nigeria	To assess the treatment practices of HWs for mRDT-negative patients in Oyo State and to identify the factors associated with prescribing antimalarial medicine to such patients	Quantitative, Questionnaire- based	318 HWs	HWs' lack of training on mRDT use and poor knowledge of causes of fever were associated with the prescription of antimalarials to mRDT- negative patients
[35]	Adherence to malaria diagnosis and treatment guidelines among healthcare workers in Ogun State, Nigeria	To determine adherence to malaria diagnosis and treatment guideline among health care service providers in public and private sectors in Ogun State, southwest, Nigeria	Quantitative, Interviewer-administered questionnaire and health facility observational checklist	216 HWs	Training on malaria case management, access to the guidelines, and availability of malaria diagnostic tools were not associated with adherence to the guidelines. The availability of recommended antimalarial medicine mainly influenced treatment prescription
[36]	Health systems readiness and quality of inpatient malaria case- management in Kano State, Nigeria	To evaluate hospital and health worker readiness for the policy implementation, health worker knowledge of case-management recommendations, and the quality of inpatient management for patients admitted with suspected malaria	Mixed method, interviews with the inpatient HWs and knowledge assessment using self-administered, multiple- choice questions	154 in-patient HWs in public hospitals	Treatment for severe malaria is compromised by a lack of malaria diagnostics, stock-outs of artesunate, and lack of supportive interventions (training and supervision) for HWs
[37]	Assessment of use of national guidelines for malaria case management among pediatric resident doctors attending an update course in Benin City, Nigeria	To assess the awareness of the Nigerian pediatric residents of the national guidelines for malaria case management, including antimalarial prescription practices for uncomplicated and severe malaria	Quantitative, self-administered questionnaire	108 pediatric resident doctors in teaching and state hospitals	Poor knowledge of and lack of formal training on the national guidelines was identified as a barrier to correctly prescribing antimalarial drugs for both uncomplicated and severe malaria
[38]	Acceptability of pre-referral rectal artesunate for severe malaria in children under 5 years by health workers and caregivers in the Democratic Republic of the Congo, Nigeria and Uganda	To provide evidence of the acceptability of rectal artesunate in communities in which it has been introduced on a large scale	Quantitative, Questionnaire- based survey	37 – 55 CHWs and health workers in primary health centers	High acceptability (98–100%) of rectal artesunate by HWs favors its use in pre- referral management of severe malaria in under-five children
